# Does Prehospital Suspicion of Sepsis Shorten Time to Administration of Antibiotics in the Emergency Department? A Retrospective Study in One University Hospital

**DOI:** 10.3390/jcm12175639

**Published:** 2023-08-30

**Authors:** Matthias Bollinger, Nadja Frère, Alexander Daniel Shapeton, Weronika Schary, Matthias Kohl, Clemens Kill, Joachim Riße

**Affiliations:** 1 Department of Anesthesiology, Intensive Care, Emergency Medicine and Pain Therapy, Schwarzwald-Baar Hospital, Klinikstrasse 11, 78052 Villingen-Schwenningen, Germany; 2 Department of Anesthesiology I, Faculty of Health, Witten/Herdecke University, 58455 Witten, Germany; 3 Center of Emergency Medicine, University Hospital Essen, 45147 Essen, Germany; 4 Department of Anesthesia, Critical Care and Pain Medicine, Boston Veterans Affairs Healthcare System, West Roxbury, MA 02132, USA; 5 Tufts University School of Medicine, Boston, MA 02111, USA; 6 Institute of Precision Medicine, Faculty of Medical and Life Sciences, Furtwangen University, 78054 Villingen-Schwenningen, Germany

**Keywords:** qSOFA, emergency medical services, screening, scoring, surviving sepsis campaign

## Abstract

Early treatment is the mainstay of sepsis therapy. We suspected that early recognition of sepsis by prehospital healthcare providers may shorten the time for antibiotic administration in the emergency department. We retrospectively evaluated all patients above 18 years of age who were diagnosed with sepsis or severe infection in our emergency department between 2018 and 2020. We recorded the suspected diagnosis at the time of presentation, the type of referring healthcare provider, and the time until initiation of antibiotic treatment. Differences between groups were calculated using the Kruskal–Wallis rank sum test. Of the 277 patients who were diagnosed with severe infection or sepsis in the emergency department, an infection was suspected in 124 (44.8%) patients, and sepsis was suspected in 31 (11.2%) patients by referring healthcare providers. Time to initiation of antibiotic treatment was shorter in patients where sepsis or infection had been suspected prior to arrival for both patients with severe infections (*p* = 0.022) and sepsis (*p* = 0.004). Given the well-described outcome benefits of early sepsis therapy, recognition of sepsis needs to be improved. Appropriate scores should be used as part of routine patient assessment to reduce the time to antibiotic administration and improve patient outcomes.

## 1. Introduction

Early treatment is one of the pillars of sepsis therapy [1], with many studies demonstrating worse outcomes when initiation of treatment is delayed [2,3]. Sepsis and its resultant long-term sequelae are a leading cause of critical illness and mortality worldwide. Additionally, sepsis and its downstream sequela are associated with high healthcare costs [4]. Given these high stakes, recent efforts have been made to identify this pathology earlier and reduce the time to initiation of antibiotic therapy. The goal of these efforts is to ultimately reduce the morbidity and mortality associated with sepsis.

Current guidelines recommend the administration of antimicrobials immediately, ideally within the first hour, in patients with possible septic shock or a high likelihood of sepsis. For patients with possible sepsis but without signs of shock, administration of antimicrobials is recommended within the first three hours after recognition [1].

To the best of our knowledge, data are unavailable on whether prehospital suspicion of sepsis—by paramedics, general practitioners, or prehospital emergency physicians—shortens the time to diagnosis of sepsis and initiation of antibiotic treatment upon arrival to a hospital. We conducted this retrospective study to investigate the suspected prehospital diagnoses for septic patients at the time of presentation to the emergency department, as well as whether suspicion of sepsis by prehospital healthcare personnel was associated with decreased time to antibiotic administration. We hypothesized that a presumed diagnosis of sepsis in the prehospital setting would be associated with a shorter time to antibiotic administration.

## 2. Material and Methods

### 2.1. Study Design

The study was designed as a retrospective cohort study and was conducted between March 2021 and October 2022 at Essen University Hospital. The University Hospital of Essen serves the Ruhr metropolis with a population of 5,147,820 (2022). The Emergency Department of the University Hospital sees 46,000 patients annually (2022), of which 11,000 are pediatric patients. We evaluated all patients who were diagnosed with sepsis or severe infection (possible sepsis) in the emergency department of our institution in the years 2018–2020 for fulfillment of inclusion criteria. The study was approved by the ethics committee of the medical faculty of the University of Duisburg-Essen on 16 September 2020 under study number 20-9550-BO and had been preregistered in the German Clinical Trials Register on 26 February 2021 under DRKS00022986. The study was carried out in accordance with all relevant regulations and as described by the study protocol. Informed consent was not deemed necessary by the ethics committee of the medical faculty of the University of Duisburg-Essen.

### 2.2. Participants

We extracted all patients who were diagnosed with sepsis during their hospital stay from our hospital information system (Medico, CompuGroup Medical SE & Co. KGaA, Koblenz, Germany). We included all patients over 18 years of age who had been diagnosed with sepsis or severe infection (possible sepsis) while in our Emergency Department. 

Sepsis was defined by the Third International Consensus Definitions on Sepsis and Septic Shock (Sepsis-3) in 2016 as a life-threatening organ dysfunction caused by a dysregulated host response to infection [4]. This is clinically operationalized by an increase in the sequential organ failure assessment (SOFA) score of 2 points or more from baseline. One of the goals of updating the definition was to establish clinical criteria that offer greater consistency for epidemiologic studies and clinical trials. However, in the setting of an emergency department, it is often unclear if an organ dysfunction had been preexisting or is a new finding. Furthermore, in an out-of-hospital setting, laboratory values are not readily available for calculating SOFA scores. Therefore, the quick sequential organ failure assessment (qSOFA) score was introduced in 2016 alongside the definition of an increase of 2 points or more in the SOFA score to help identify patients with poor outcomes early using bedside criteria. The score consists of 3 variables: altered mentation with a Glasgow coma scale of less than 15, hypotension with a systolic blood pressure of 100 mmHg or less, and respiratory distress with a respiratory rate of 22/min or greater. A qSOFA-score of 2 or 3 in patients with suspected infection is equivalent to an increase of 2 points in the SOFA score to fulfill sepsis criteria, according to the Sepsis-3 Definition [4]. The qSOFA score has a high prognostic value for poor outcomes but is less useful as a screening tool for sepsis, although it is often used for this purpose. In 2021, the surviving sepsis campaign recommended against the use of the qSOFA score alone as a screening tool for sepsis because of its poor sensitivity [1]. Furthermore, a distinction was made in the surviving sepsis campaign guidelines of 2021 between patients with septic shock or high likelihood of sepsis and patients with possible sepsis but without signs of shock with regard to time until antimicrobials should be administered [1]. Because of the limitations of the qSOFA score for identifying patients with sepsis, we decided to define sepsis as having the diagnosis of sepsis established at the time of admission to the hospital from the emergency department, while severe infection (possible sepsis) was similarly defined as having established the diagnosis at the time of admission. These definitions were used independent of the results of qSOFA or other scores. Patients who did not meet inclusion criteria were excluded from the study.

### 2.3. Measurements

We collected data from patients who had been diagnosed with sepsis or severe infection (possible sepsis) in our emergency department. Patient data were extracted from the electronic medical records of our emergency department (ERPath, eHealth-Tec Innovations GmbH, Berlin, Germany) and from our hospital information system. We recorded whether the patient was sent or brought to the hospital by a general practitioner, paramedics, a prehospital emergency physician, or presented by themselves. We also recorded the suspected diagnoses at the time of presentation and the time of initiation of antibiotic treatment as documented by the time the emergency physician created the electronic prescription. We also collected gender, age, and vital signs data at the time of presentation to the emergency department (systolic blood pressure, respiratory rate, Glasgow coma scale) to calculate the quick sequential organ failure assessment (qSOFA) score.

### 2.4. Outcomes

The primary endpoint was the suspected prehospital diagnoses at the time of emergency department arrival in patients who were later diagnosed with sepsis. The secondary endpoint was the time to antibiotic treatment.

### 2.5. Statistical Analysis

The data were collected and transferred to a spreadsheet in Excel (Excel, Microsoft Corporation, Redmond, WA, USA). Statistical analysis was performed with R (R Core Team 2022, Vienna, Austria). Patient characteristics were described using the mean, standard deviation (SD), median, and interquartile range (IQR). 

Fisher’s exact test was used to determine differences between suspected diagnoses and the results of qSOFA-scoring. Frequencies of suspected diagnoses and referring healthcare providers were described using total numbers and percentages. The time to initiation of antibiotic treatment was described using the median and IQR. Differences between groups were calculated using the Kruskal–Wallis rank sum test. A *p*-value < 0.05 was considered significant.

## 3. Results

Of 362 patients diagnosed with sepsis during their hospital stay (discharge diagnosis from the hospital), 277 had a severe infection (possible sepsis) or confirmed sepsis when they were admitted to the hospital by the emergency department and were included for further analysis. Of those 277 patients, 161 had a severe infection, and 116 were diagnosed with sepsis in the emergency department.

Gender distribution was not equal, with 120 female (43.3%) and 157 male (56.7%) participants. In total, 70 female participants and 91 male participants were diagnosed with a severe infection. Moreover, 50 female and 66 male participants were diagnosed with sepsis.

The mean ages were similar in patients with severe infection, 70 years (SD 16.1, median 73.0, IQR 20.00) and sepsis 74 years (SD 14.6, median 76.5, IQR 18.25).

### 3.1. Diagnoses in the Emergency Department at Time of Hospital Admission

**Figure 1:**
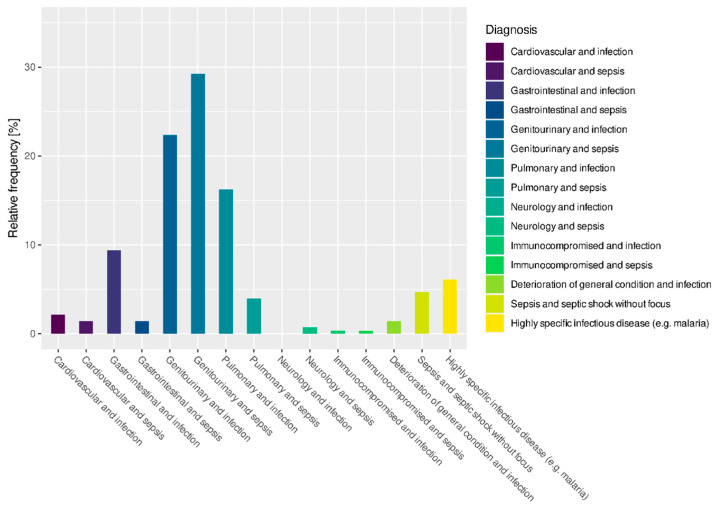
Frequency of diagnoses in the emergency department at the time of hospital admission (%).

Figure 1 shows the frequency of diagnoses that were made in the emergency department at the time of hospital admission.

### 3.2. Results of qSOFA Scoring in the Patient Cohort

Because of missing values, we were only able to calculate the qSOFA in 103 out of 161 patients (64.0%) who were diagnosed with a severe infection and 80 out of 116 patients (69.0%) who were diagnosed with sepsis. We calculated the qSOFA scores from data that were taken at the time of presentation to the emergency department in order to investigate the patient’s status as seen by the referring prehospital healthcare provider. The vital parameter most often missing for calculation of the qSOFA score was the Glasgow coma scale (missing in 26/116 patients with sepsis and in 47/161 patients with a severe infection), followed by respiratory rate (missing in 12/116 patients with sepsis and in 14/161 patients with a severe infection) and the systolic blood pressure (missing in 1/116 patient with sepsis and in 2/161 patients with a severe infection.

Of the 161 patients with a severe infection, 35/103 (34.0%) had a qSOFA of 0, 35/103 (34.0%) had a qSOFA of 1, 30/103 (29.1%) had a qSOFA of 2, and 3/103 (2.9%) had a qSOFA of 3. Of the 116 patients that were diagnosed with sepsis, 14/80 (17.5%) had a qSOFA of 0, 24/80 (30.0%) had a qSOFA of 1, 32/80 (40.0%) had a qSOFA of 2, and 10/80 (12.5%) had a qSOFA of 3.

While the qSOFA scores of patients who were diagnosed with sepsis differed significantly from those patients who were diagnosed with a severe infection (*p* = 0.007) and time to initiation of antibiotic treatment differed between qSOFA-Scores (*p* < 0.001), there was no significant difference in the qSOFA scores between the different suspected diagnoses made by prehospital healthcare providers (*p* = 0.540).

### 3.3. Referring Healthcare Personnel

The largest proportion of patients were presented to the emergency department by paramedics (106/277, 38.3%). Prehospital emergency physicians presented 80/277 patients (28.9%), while 36/277 patients (13.0%) were referred by their general practitioner. Meanwhile, 39/277 patients (14.1%) presented themselves, and in 16/277 patients (5.8%), it was not recorded whether the patient was referred by a healthcare provider.

### 3.4. Suspected Diagnoses at Time of Presentation

**Figure 2:**
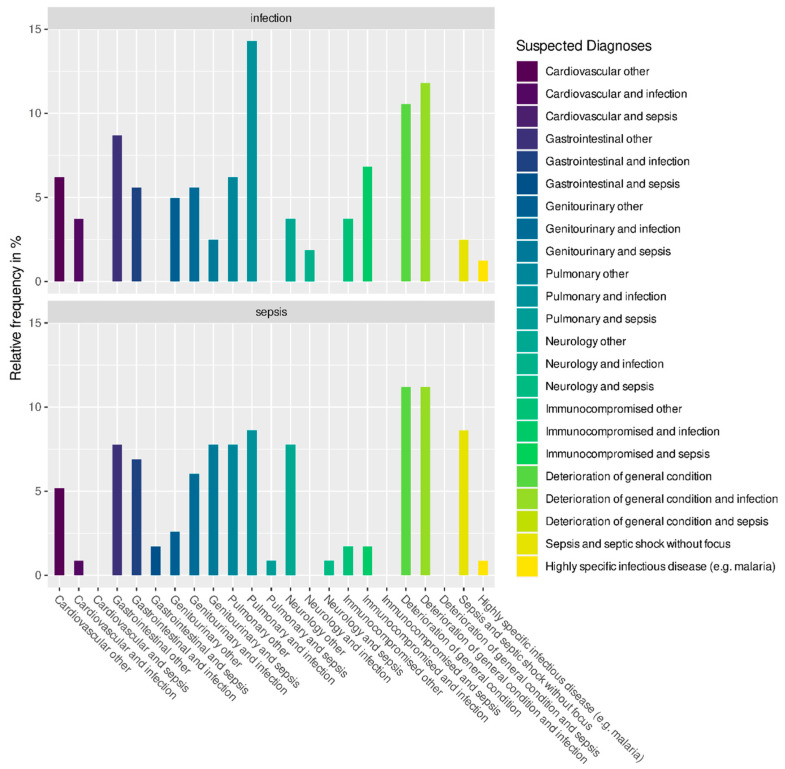
Suspected diagnoses made by prehospital healthcare providers for patients who were diagnosed with sepsis or severe infection in the emergency department (%).

An infection was suspected in 124/277 (44.8%) patients, and sepsis was suspected in 31/277 (11.2%) patients by the referring healthcare provider. Moreover, 122/277 patients (44.0%) were presented to the emergency department with other suspected diagnoses. Figure 2 shows the suspected diagnoses in detail for patients who were diagnosed with sepsis or severe infection in the emergency department.

### 3.5. Frequency of Suspected Sepsis or Infection by Different Referring Healthcare Providers

Table 1 demonstrates the suspected diagnoses organized by type of referring provider.

### 3.6. Time to Initiation of Antibiotic Treatment in Patients Diagnosed with a Severe Infection

**Figure 3:**
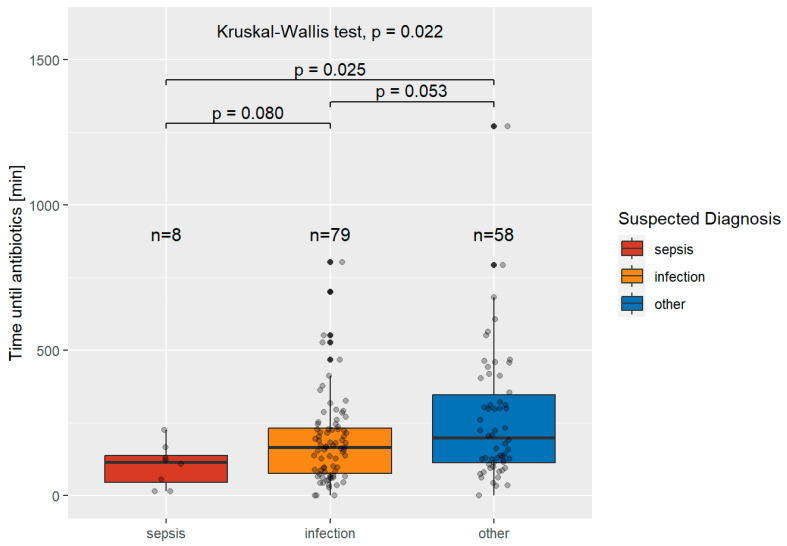
Time to initiation of antibiotic, in minutes, in patients with severe infection and the suspected diagnoses of their prehospital healthcare personnel.

Figure 3 shows the time to initiate antibiotic treatment in patients diagnosed with a severe infection in the emergency department and the suspected diagnoses at the time of presentation to the Emergency Department.

The median times were 115.5 min (IQR 92.25) for suspected sepsis, 167 min (IQR 155.00) for patients with suspected infection, and 198.5 min (IQR 232.50) for other suspected diagnoses. The differences between these groups were significant (*p* = 0.022).

Time to initiation of antibiotic treatment was not documented in 16/161 patients with severe infections (of these, 3 were suspected infections and 13 cases were of other suspected diagnoses).

### 3.7. Time to Initiation of Antibiotic Treatment in Patients Diagnosed with Sepsis

**Figure 4:**
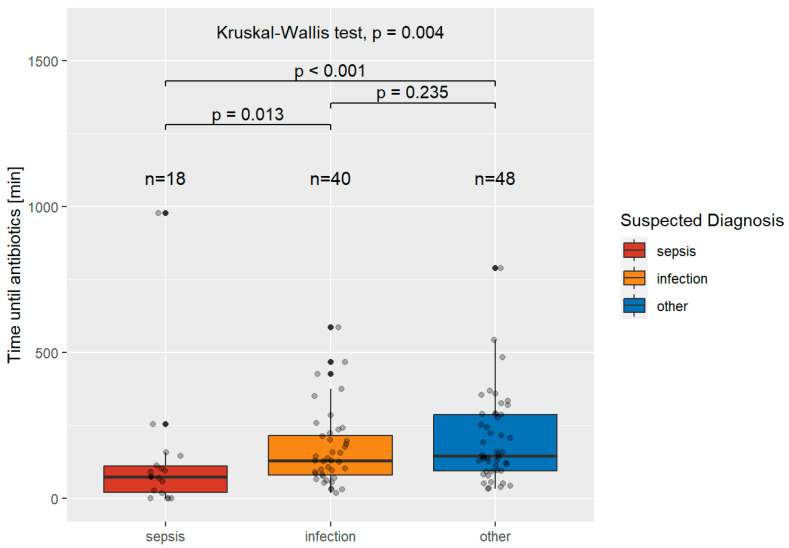
Time to initiation of antibiotic treatment, in minutes, in patients with sepsis and the suspected diagnoses by their prehospital healthcare personnel.

Figure 4 shows the time to initiation of antibiotic treatment in patients who were diagnosed with sepsis in the emergency department and their suspected diagnoses at the time of presentation.

The median times were 74.5 min (IQR 89.25) for suspected sepsis, 129.5 min (IQR 136.25) for patients with suspected infection, and 145.5 min (IQR 191.75) for other suspected diagnoses. The differences between the groups were significant (*p* = 0.004). Time to initiation of antibiotic treatment was not documented in 10/116 patients with sepsis (of which there were 5 cases of suspected sepsis, 2 cases of suspected infection, and 3 cases of other suspected diagnoses).

### 3.8. Time to Initiation of Antibiotic Treatment Stratified by Referring Healthcare Providers

**Figure 5:**
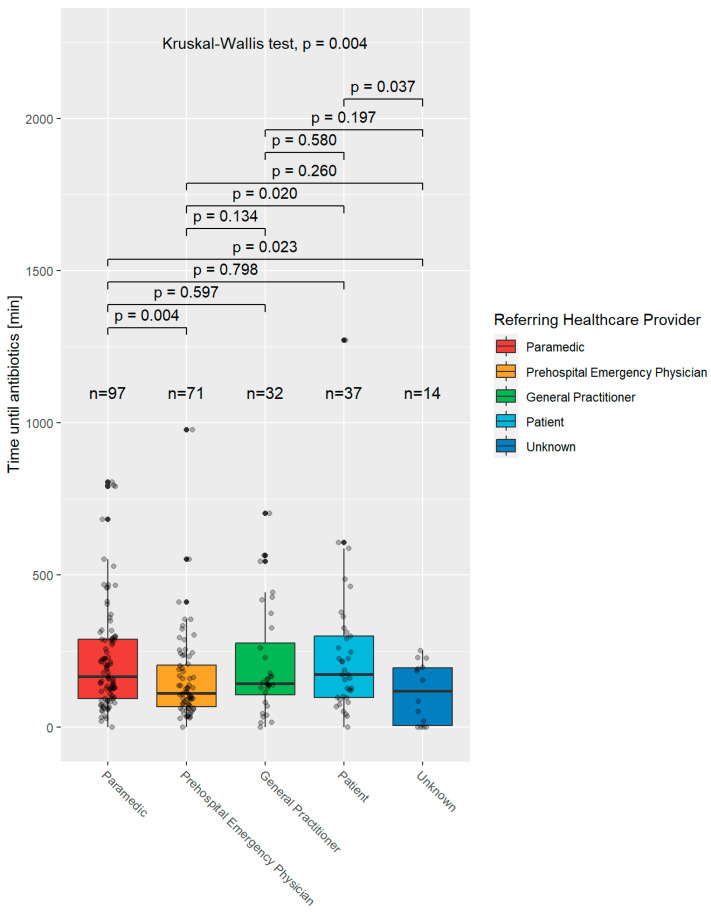
Time to initiation of antibiotic treatment, in minutes, in patients with severe infection or sepsis and their referring/presenting healthcare provider.

Figure 5 shows the time to initiation of antibiotic treatment in minutes in patients with severe infections or sepsis and which healthcare provider referred or presented the patient to the emergency department.

The median times were 167.0 min (IQR 194.0) for patients presented by paramedics, 111.0 min (IQR 137.5) for patients presented by a prehospital emergency physician, 144.5 min (IQR 170.5) for patients referred by their general practitioner, 173.0 min (IQR 203.0) for self-presenters and 119.5 min (190.5) in patients where no healthcare provider was recorded. Differences between the different referring healthcare providers were significant (*p* = 0.004).

In 9/106 patients presented by paramedics, 9/80 patients presented by prehospital emergency physicians, 4/32 patients referred by general practitioners, 2/39 self-presenters, and 2/16 patients where no referring healthcare provider was recorded, time to administration of antibiotics was not recorded.

## 4. Discussion

Our main findings were:

1. Infection was suspected in 124/277 (44.8%) patients, and sepsis was suspected in 31/277 (11.2%) patients by the referring healthcare provider in patients later diagnosed with sepsis or severe infection (possible sepsis) in the emergency department. In total, 122/277 patients (44.0%) later diagnosed with sepsis or severe infection (possible sepsis) were presented with other suspected diagnoses.
2. There was no significant difference in qSOFA scores between the different suspected diagnoses made by prehospital healthcare providers (*p* = 0.540). However, qSOFA scores of patients who were later diagnosed with sepsis in the emergency department differed significantly from patients who were diagnosed with a severe infection (*p* = 0.007).
3. Time to initiation of antibiotic treatment was shorter in patients where sepsis or infection had been suspected prior to arrival for both patients diagnosed with severe infections (*p* = 0.022) and sepsis (*p* = 0.004) in the emergency department.

Rapid recognition and early treatment are the mainstays of sepsis therapy [1]. However, as there is no gold standard test for sepsis and laboratory tests are usually not available in the prehospital setting, making the diagnosis can be challenging. In our study, most patients with sepsis or severe infections were presented to the Emergency Department by paramedics without assessment by a prehospital emergency physician. However, of 106 patients with sepsis or severe infection brought in by paramedics, sepsis was only suspected in 4 patients (3.8%), and an infection was only suspected in 49 patients (46.2%). In 53 patients (43.4%) later diagnosed with sepsis or a severe infection, this was not recognized or suspected at the time of presentation. Prehospital emergency physicians performed better, suspecting the diagnosis of sepsis in 16 of 80 patients (20%). Germany has a two-tiered emergency medical services system with paramedics and prehospital emergency physicians working together. Emergency call-takers decide whether a paramedic ambulance is deployed and whether a rapid response vehicle with a prehospital emergency physician is also deployed simultaneously. This determination is made based on a catalog of standard complaints and mechanisms of injury. Furthermore, paramedics are expected to call for backup from a prehospital emergency physician if the severity of the illness or the procedures needed to stabilize the patient exceed paramedic training. Prehospital emergency physicians work primarily in the hospital (e.g., in anesthesiology, intensive care, and emergency department) and are assigned to routine shifts on the rapid response vehicle. The better performance of prehospital emergency physicians demonstrated in our data may, therefore, be attributable to a higher level of awareness due to treating these patients more routinely in the hospital setting. On the other hand, prehospital emergency physicians are usually deployed to care for sicker patients and, therefore, might be biased toward making these diagnoses more often. That said, despite their additional training and potential familiarity, prehospital emergency physicians also fail to make the diagnosis in 38/80 (47.5%) patients. Self-presenters did not suspect sepsis in any case but had the highest suspicion of an infection (26 of 39 patients, 66.6%). This finding is not surprising given that “sepsis” is largely a medical term with which most lay people would not be familiar. It should be noted that of the 277 patients diagnosed with severe infection or sepsis, only 31 patients (11.2%) presented with suspected sepsis, and only 124 patients (44.8%) were suspected to have an infection. In 122 patients (44%) later diagnosed with severe infection or sepsis in the emergency department, this was not recognized by prehospital personnel.

In recent years, efforts have been made to raise awareness of sepsis in the prehospital field, but consistent recognition continues to lag. Current guidelines for the management of sepsis and infection do not adequately take prehospital care into account—though early treatment must, of course, begin with early recognition, and early recognition begins in the prehospital setting. In this study, time to antibiotic treatment differed significantly depending on the diagnoses that were suspected at the time of presentation to the Emergency department in both septic patients (*p* = 0.004) and patients with severe infections (*p* = 0.022) and between the different referring healthcare providers (*p* = 0.004).

Patients who were diagnosed with sepsis in the emergency department and in whom sepsis was suspected at the time of presentation received antibiotic treatment in 74.5 (IQR 89.25) minutes, while patients with severe infections and suspected sepsis received treatment in 115.5 (IQR 92.25) minutes. Patients who were diagnosed with a severe infection in the emergency department and in whom sepsis was suspected at the time of presentation received treatment after 129.5 (IQR 136.25) minutes, while patients with suspected infection received treatment after 167 (IQR 155.00) minutes. These findings stand in stark contrast to those in septic patients where neither sepsis nor infection was suspected, who experienced a prolonged time to antibiotic treatment of 145.5 min (IQR 191.75), as well as patients with a severe infection where neither sepsis nor infection was suspected, who experienced a time to antibiotic treatment of 198.5 min (IQR 232.50).

In this study, we identified statistically significant differences between initiation of antibiotic therapy based on referring healthcare providers (*p* = 0.004). Patients who were presented by prehospital emergency physicians received treatment within 111.0 min (IQR 137.5), patients who were referred by their general practitioner received treatment within 144.5 min (IQR 170.5), self-presenters received treatment within 173.0 min (IQR 203.0), while patients presented by paramedics received antibiotics after 167.0 min (IQR 194.0). Though we are unable to draw firm conclusions from this data due to uncontrolled confounders such as bias in deployments, our findings do suggest that further investigation is warranted into the different providers who manage sepsis in the prehospital setting.

Our data shows that early recognition of sepsis or severe infection by the referring healthcare providers is associated with a shorter time for effective treatment in the hospital. This might reflect that sepsis or severe infection is earlier recognized in sicker patients, as patients who were diagnosed with sepsis in the emergency department received antibiotics earlier than patients with severe infections. However, our data shows that in septic patients, as well as in patients with severe infections, the time to antibiotics differed significantly depending on the suspected diagnoses, while qSOFA scores did not differ significantly between the different suspected diagnoses (*p* = 0.540).

These findings have significant implications with regard to patient assessment in the prehospital field as well as in the emergency department. To make the diagnosis, it is critical to have a high suspicion of the illness and to use appropriate screening tools routinely. Several screening tools are available, but the diagnostic accuracy of these tests is variable, with most either having low sensitivity and/or poor prognostic value, such as the systemic inflammatory response syndrome criteria [5,6]. Furthermore, as most scoring tools require laboratory values, they may not be useful for out-of-hospital care. In 2016, the qSOFA score was introduced [4] as a better prognostic tool for associated mortality [7]. Unfortunately, the qSOFA score has a low sensitivity and is therefore not useful as a screening tool [8]. That said, the score is still widely used by clinicians for this purpose, with many of them unaware of its limitations. One reason might be that the score is easy to remember and convenient to calculate from parameters that are collected routinely during patient assessment with no laboratory values needed. The updated surviving sepsis campaign 2021 guidelines strongly recommend against the use of qSOFA as a single tool for the identification of sepsis, while other scores, such as the National Early Warning Score (NEWS), are more sensitive for detecting critically ill patients but are significantly more complicated to calculate [8,9]. However, despite these limitations, the use of scoring systems has shown to be superior to clinical judgment alone [10]. Therefore, efforts should be made to identify a score that is sensitive enough, hence practical for use in the prehospital setting. Alternatively, as tablet computers are increasingly used by emergency medical service agencies for electronic patient care reporting, these could be programmed to calculate scores automatically from the patient’s vital signs when entered into the electronic patient care reporting system.

In the emergency department, it may be possible to calculate these scores during triage, as many hospital systems take the patient’s vital signs at this time. An automated calculation could also potentially be employed in this setting, as many hospitals have established electronic patient records, and vital signs are often automatically transferred, thus not prolonging the triage process. That said, predictive scoring results in many scenarios that are only one piece of the clinical puzzle—carrying with them the potential to harm patients if algorithms are followed blindly and the individual patient and circumstances are not considered [11,12].

Another potential area for improvement could be the acceleration of emergency management in patients where the diagnosis of sepsis is suspected by prehospital healthcare providers. In our study, patients who were diagnosed with sepsis in the emergency department and who had been presented with suspected sepsis received antibiotic treatment after 74.5 min (IQR 89.25). Even with the advantage of the prehospital suspected diagnosis, this exceeds the recommendation of one hour for septic patients [1]. However, as there is no gold-standard test for sepsis, this delay could, in part, reflect waiting times for things such as laboratory investigations. Point-of-care testing for markers of an infection and organ dysfunction could potentially reduce waiting times and help facilitate earlier diagnosis [13]. Given the rapid improvements in point-of-care diagnostics, particularly during the COVID-19 pandemic, with devices becoming smaller, more portable, and less expensive [14], these devices could support clinicians in making earlier and more accurate prehospital diagnoses. This idea has already been tested and operationalized with prehospital lactate measurement [15,16].

However, it is unclear if changes in testing would improve outcomes compared to lowering the threshold for administration of a first dose of antibiotics in suspected sepsis. Antibiotics could more routinely be administered in the prehospital setting. A recent meta-analysis found a significant reduction in the 28-day mortality rate when patients received a first dose of a broad-spectrum antibiotic in the prehospital field compared to standard care [17]. An acceleration of the management of patients where the diagnosis has already been made is probably the easiest way to further improve outcomes. However, for this to be impactful, the diagnosis has to be suspected early.

## 5. Limitations

The study retrospectively evaluates suspected diagnoses, type of referring healthcare provider, and time to initiation of antibiotic treatment in patients who were diagnosed with sepsis and severe infection in the emergency department of one university hospital. Therefore, the findings of this study might be of limited value to other populations, as the training of different healthcare providers might differ between different services and across borders. Our study is also subject to the usual biases and limitations associated with a retrospective design.

We only evaluated patients who were diagnosed with severe infection or sepsis. Therefore, we were not able to evaluate how often patients were presented to the emergency department with suspected sepsis or severe infection and were diagnosed with other illnesses.

We were only able to calculate qSOFA scores in 103 out of 161 patients (64.0%) who were diagnosed with a severe infection and 80 out of 116 patients (69.0%) who were diagnosed with sepsis because we only calculated qSOFA scores from vital parameters that were recorded at presentation to the emergency department. Utilizing vital parameters from later in the patient’s stay would have confounded our data due to potential deterioration in these parameters due to illness progression.

Time to initiation of antibiotic treatment was extracted from the electronic healthcare record of the emergency department from the time of administrative admission of the case file in the emergency department to the time when the care-taking emergency physician prescribed antibiotics via the electronic Emergency Department Management Program. The true time to initiation of antibiotic treatment might differ from the point in time when the treatment was documented in the healthcare record. This may be particularly relevant in critically ill patients where treatment begins immediately, and documentation often follows later.

## 6. Conclusions

Early recognition of sepsis in the prehospital setting needs to be improved. Our study demonstrates that prehospital recognition has the potential to shorten the time to antibiotic treatment upon arrival to the hospital, and this may translate to meaningful improvements in patient outcomes. Appropriate scoring systems must be routinely used in patient assessment to aid effective prehospital recognition.

## Figures and Tables

**Table 1:** Suspected diagnoses organized by referring healthcare provider (total number and %).

Suspected Diagnoses/ Referring Healthcare Provider	Infection n (% of Diagnoses by Respective Profession)	Sepsis n (% of Diagnoses by Respective Profession)	Other n (% of Diagnoses by Respective Profession)
Paramedics	49/106 (46.2%)	4/106 (3.8%)	53/106 (50.0%)
Prehospital Emergency Physicians	26/80 (32.5%)	16/80 (20.0%)	38/80 (47.5%)
General Practitioner	15/36 (41.7%)	5/36 (13.9%)	16/36 (44.4%)
Self-referring	26/39 (66.7%)	0/39 (0.0%)	13/39 (33.3%)
Unknown	8/16 (50.0%)	6/16 (37.5%)	2/16 (12.5%)

## Data Availability

The datasets used and/or analyzed during the current study are available from the corresponding author upon reasonable request.

## References

[1] Evans L., Rhodes A., Alhazzani W., Antonelli M., Coopersmith C.M., French C., Machado F.R., Mcintyre L., Ostermann M., Prescott H.C. (2021). Surviving sepsis campaign: International guidelines for management of sepsis and septic shock 2021. Intensiv. Care Med..

[2] Kumar A., Roberts D., Wood K.E., Light B., Parrillo J.E., Sharma S., Suppes R., Feinstein D., Zanotti S., Taiberg L. (2006). Duration of hypotension before initiation of effective antimicrobial therapy is the critical determinant of survival in human septic shock*. Crit. Care Med..

[3] Ferrer R., Martin-Loeches I., Phillips G., Osborn T.M., Townsend S., Dellinger R.P., Artigas A., Schorr C., Levy M.M. (2014). Empiric Antibiotic Treatment Reduces Mortality in Severe Sepsis and Septic Shock From the First Hour: Results from a guideline-based per-formance improvement program. Crit. Care Med..

[4] Singer M., Deutschman C.S., Seymour C.W., Shankar-Hari M., Annane D., Bauer M., Bellomo R., Bernard G.R., Chiche J.-D., Coopersmith C.M. (2016). The Third International Consensus Definitions for Sepsis and Septic Shock (Sepsis-3). JAMA.

[5] Churpek M.M., Zadravecz F.J., Winslow C., Howell M.D., Edelson D.P. (2015). Incidence and Prognostic Value of the Systemic Inflammatory Response Syndrome and Organ Dysfunctions in Ward Patients. Am. J. Respir. Crit. Care Med..

[6] Kaukonen K.-M., Bailey M., Pilcher D., Cooper D.J., Bellomo R. (2015). Systemic Inflammatory Response Syndrome Criteria in Defining Severe Sepsis. New Engl. J. Med..

[7] Vincent J.-L., de Mendonca A., Cantraine F., Moreno R., Takala J., Suter P.M., Sprung C.L., Colardyn F., Blecher S. (1998). Use of the SOFA score to assess the incidence of organ dysfunction/failure in intensive care units: Results of a multicenter, prospective study. Working group on “sepsis-related problems” of the European Society of Intensive Care Medicine. Crit. Care Med..

[8] Usman O.A., Usman A.A., Ward M.A. (2019). Comparison of SIRS, qSOFA, and NEWS for the early identification of sepsis in the Emergency Department. Am. J. Emerg. Med..

[9] Wattanasit P., Khwannimit B. (2021). Comparison the accuracy of early warning scores with qSOFA and SIRS for predicting sepsis in the emergency department. Am. J. Emerg. Med..

[10] Fullerton J.N., Price C.L., Silvey N.E., Brace S.J., Perkins G.D. (2012). Is the Modified Early Warning Score (MEWS) superior to clinician judgement in detecting critical illness in the pre-hospital environment?. Resuscitation.

[11] Sutton N.R., Gurm H.S. (2015). Door to Balloon Time: Is There a Point That Is Too Short?. Prog. Cardiovasc. Dis..

[12] Ortoleva J.P., Cordes C.L., Salehi P., Shapeton A.D. (2021). Predictive Scoring: Should It Tell Us the Odds?. J. Cardiothorac. Vasc. Anesth..

[13] Pfäfflin A., Schleicher E. (2008). Inflammation markers in point-of-care testing (POCT). Anal. Bioanal. Chem..

[14] Schary W., Paskali F., Rentschler S., Ruppert C., Wagner G.E., Steinmetz I., Deigner H.-P., Kohl M. (2022). Open-Source, Adaptable, All-in-One Smartphone-Based System for Quantitative Analysis of Point-of-Care Diagnostics. Diagnostics.

[15] Boland L., Hokanson J., Fernstrom K., Kinzy T., Lick C., Satterlee P., LaCroix B. (2016). Prehospital Lactate Measurement by Emergency Medical Services in Patients Meeting Sepsis Criteria. West. J. Emerg. Med..

[16] Guerra W.F., Mayfield T.R., Meyers M.S., Clouatre A.E., Riccio J.C. (2013). Early Detection and Treatment of Patients with Severe Sepsis by Prehospital Personnel. J. Emerg. Med..

[17] Varney J., Motawea K.R., Kandil O.A., Hashim H.T., Murry K., Shah J., Shaheen A., Akwari J., Awad A.K., Rivera A. (2022). Prehospital administration of broad-spectrum antibiotics for sepsis patients: A systematic review and meta-analysis. Health Sci. Rep..

